# T-cell activation promotes tumorigenesis in inflammation-associated cancer

**DOI:** 10.1186/1742-4690-6-116

**Published:** 2009-12-17

**Authors:** Dan Rauch, Shimon Gross, John Harding, Sirosh Bokhari, Stefan Niewiesk, Michael Lairmore, David Piwnica-Worms, Lee Ratner

**Affiliations:** 1 Department of Medicine, Division of Molecular Oncology, Washington University School of Medicine, St Louis, MO 63110, USA; 2 Molecular Imaging Center, Mallinckrodt Institute of Radiology, Washington University School of Medicine, St Louis, MO 63110, USA; 3 College of Veterinary Medicine, Department of Veterinary Biosciences, The Ohio State University, Columbus, OH 43210, USA; 4 Center for Retrovirus Research, Department of Veterinary Biosciences, The Ohio State University, Columbus, OH 43210, USA; 5 Department of Molecular Biology and Pharmacology, Washington University School of Medicine, St Louis, MO 63110, USA

## Abstract

Chronic inflammation has long been associated with a wide range of malignancies, is now widely accepted as a risk factor for development of cancer, and has been implicated as a promoter of a variety of cancers including hematopoietic malignancies. We have described a mouse model uniquely suited to examine the link between inflammation and lymphoma in which the Tax oncogene, expressed in activated T and NK cells, perpetuates chronic inflammation that begins as microscopic intraepithelial lesions and develops into inflammatory nodules, subcutaneous tumors, and large granular lymphocytic leukemia. The use of bioluminescent imaging in these mice has expanded our ability to interrogate aspects of inflammation and tumorigenesis non-invasively. Here we demonstrate that bioluminescence induction in these mice correlated with inflammation resulting from wounding, T cell activation, and exposure to chemical agents. In experiments in which long-term effects of inflammation on disease outcome were monitored, the development of lymphoma was promoted by an inflammatory stimulus. Finally we demonstrated that activation of T-cells in T-cell receptor (TCR) transgenic TAX-LUC animals dramatically exacerbated the development of subcutaneous TCR^- ^CD16^+ ^LGL tumors. The role of activated T-cells and acquired immunity in inflammation-associated cancers is broadly applicable to hematopoietic malignancies, and we propose these mice will be of use in dissecting mechanisms by which activated T-cells promote lymphomagenesis *in vivo*.

## Background

Malignant transformation of the cancer cell is promoted and often preceded by changes in the tumor microenvironment, rich in inflammatory cells, growth factors, and DNA damage promoting agents. A wide range of malignancies are promoted by chronic inflammation associated with chemical, physical, or microbial factors [1-4]. The diversity of oncogenic factors associated with inflammation highlights the importance of characterizing those common to a wide range of malignancies. The cellular effectors, signaling pathways, and secreted regulators involved in chronic inflammation are the soil in which the seeds of these cancers are initiated.

T-cells are central regulators of the immune response; T-cells are recruited to sites of chronic inflammation, and the infiltration of T-cells within the tumor is a critical determinant of neoplastic outcome. Naïve CD4^+ ^T-cells, or T-helper cells, that have not previously encountered an antigen differentiate into one of four committed lineages (T_H_1, T_H_2, T_H_17, T_reg_) in response to antigen presenting cells [5-10]. Conventionally, T_H_1 and T_H_2 cells promote the elimination of intracellular and extracellular pathogens respectively. More recently T_H_17 cells have been characterized for their ability to promote inflammation by recruiting neutrophils to peripheral tissues to remove extracellular pathogens, while T_reg _cells repress inflammation to keep immune hyperactivity in check. While there is no question that T-cells are recruited to sites of chronic inflammation, it is unclear whether activated T-cells promote or restrict malignancies *in vivo*.

Molecular pathways often involved in inflammation-associated tumorigenesis include JNK, STAT3, HIF-1, and nuclear factor κB (NFκB) signaling, and generation of reactive oxygen species [1,3,11,12]. These pathways are interrelated and signaling through NFκB serves as a master regulator. NFκB signaling during tumorigenesis prevents apoptosis and promotes proliferation, metastasis, and angiogenesis [13]. NFκB is activated in T-lymphocytes after T-cell receptor (TCR) engagement, as well as in other cell types through activation of toll-like receptors (TLR) [11,14,15]. NFkB is over-expressed in a wide range of malignancies, particularly cancers refractory to chemotherapy [16,17].

Soluble mediators of migration, proliferation, and signaling pathways of cells in the tumor microenvironment include cytokines and chemokines. The balance of cytokines produced in a tumor regulates the type and extent of inflammatory infiltrate, the level of cytotoxicity and genetic instability, the degree of neovascularization, and the innate and adaptive immune responses to the tumor [14,16,17].

We have developed and characterized a triple transgenic mouse model of inflammation-associated cancer that allows us to experimentally activate T cells and NFkB signaling pathways prior to the onset of tumorigenesis and to non-invasively monitor inflammation and tumor progression using bioluminescent imaging (BLI). The first transgene expresses the human T-cell leukemia virus type 1 (HTLV-1) Tax oncogene under the granzyme B promoter (GZB), which restricts expression to activated T- and NK- cells [18,19]. In activated T- and NK- cells of these mice, Tax constitutively activates both the canonical and non-canonical pathways of NFkB [20]. Moreover, tumors that arise in GZB-TAX mice are composed of malignant CD16^hi ^large granular lymphocytes (LGLs), infiltrating CD16^lo ^neutrophils, and CD16^- ^T- and B- lymphocytes [18,20-25]. Moreover, Tax stimulates and recruits inflammatory cells through induction of IFN-gamma, IL-1, IL-6, GM-CSF, RANK ligand, and TNFα [21,24,26].

The second transgene expresses firefly luciferase (LUC) under the regulation of the HTLV-1 LTR. When mice carry both the LTR-LUC and GZB TAX transgenes (TAX-LUC mice), the events associated with the expression of Tax, including T-cell activation, constitutive NFKB activation, and spontaneous tumorigenesis, can be monitored non-invasively by BLI. In these mice, inflammation was closely correlated with lymphomagenesis, and sensitive imaging technology enabled us, for the first time, to identify all stages in spontaneous tumor development including primary microscopic lesions, pre-malignant inflammatory nodules, localized tumors, and disseminated disease [25]. Thus in TAX-LUC mice, Tax expressed in mature lymphocytes activates luciferase expression which is detected non-invasively using D-luciferin as a substrate for BLI. Moreover, we recently described the use of luminol to monitor neutrophil myeloperoxidase activity, using the same imaging modality, as an independent reporter for tumor associated inflammation [27].

The third transgene is a genetic manipulation of the T-cell receptor that restricts its recognition to ovalbumin such that activation of T- cells in TCR transgenic mice can be experimentally induced by administration of ovalbumin. The majority of circulating T- cells are activated in TCR-OVA transgenic animals upon administration of ovalbumin [6,7]. The combination of these three transgenes and the properties of the oncoprotein Tax, gave us the ability to activate T cells, stimulate NFkB pathways, promote inflammation, and image these processes non-invasively using luciferase mediated BLI. We used this model to determine whether activated T- cells promote or suppress tumorigenesis *in vivo*. We discovered that the activation of T- cells in triple transgenic mice dramatically exacerbated tumor development and the onset and dissemination of LGL lymphoma. We propose that these findings are applicable to many forms of hematologic malignancy especially those associated with constitutive activation of NFkB and chronic inflammation. We further propose that this animal model will be a broadly useful tool in the delineation of the mechanisms by which T-cells promote tumorigenesis *in vivo*.

## Methods

### Transgenic Mice

Individual strains of transgenic mice utilized in this report have been previously described. In LTR-LUC, the 0.7 Kb XhoI-HindIII 5'LTR fragment of pHTE-1 drives firefly luciferase (pGL-3; Promega) [25]. In GZB-TAX, HTLV-1 Tax is regulated by the 5' flanking region (-1170 to +36) of the human granzyme B gene [18]. Mice were housed under pathogen free conditions and animal protocols were approved by the Animal Studies Committee in accordance with the guidelines of the Washington University School of Medicine.

### Flow Cytometry

Cell suspensions derived from organs or tumors were stained with FITC-conjugated FcγR II/III antibodies (clone 2.4G2; BD Pharmingen) for 30 minutes at 4°C and analyzed on a FACScan (Becton Dickinson). In three color experiments, cells were incubated with unlabelled FcγR II/III antibodies for 30 minutes to block free surface FcγR, and counterstained with PE-conjugated antibodies against TCR^ova ^(clone KJ1-26; eBioscience) and PE-Cy5 conjugated anti-CD4 (cloneGK1.5 eBioscience).

### Imaging

The IVIS100 system (Xenogen) was used to image bioluminescence in anesthetized mice (isoflurane inhalation). Standard imaging parameters included D-luciferin dose 15 mg i.p; luminol dose 200 mg/kg i.v; exposure 300 sec; binning 4; f/stop 1; no optical filter. When luminol and D-luciferin images were obtained from the same animal, the first substrate was allowed to clear for 24 hours prior to injection with the second. When necessary, hair was removed by shaving or depiliation prior to imaging. Color scale unless otherwise indicated is ×10^4 ^photons/sec/cm^2^/sr. The indicated agents were injected ip at the following dosages: con A, 2.5 mg/kg; LPS, 2.5 mg/kg; CFA, 100 μl in 100 μl PBS; poly(I:C), 1 mg/kg. For experiments involving BrdU, animals were injected with 1 mg BrdU, i.p. (BD Pharmingen) 24 hours prior to necropsy.

### Histology

Histology was performed as described [25]. Briefly, tissues were fixed in 4% paraformaldehyde and embedded in paraffin for serial sectioning. The primary BrdU antibody (Dako clone Bu20a) was used at a dilution of 1:150. The biotinylated primary antibody was incubated for 1 hour and labeled streptavidin applied for 30 minutes. Slides were developed with DAB chromogen then counterstained in Richard Allen hematoxylin. Sections were visualized with a Nikon Eclipse E400 microscope and digital images were obtained using a Magnafire camera and software (Optronics).

## Results

### Imaging Inflammation and Tumorigenesis in vivo

TAX-LUC mice are doubly transgenic mice in which i) the Tax gene from HTLV-1 is restricted to activated NK and T cells by the granzyme B promoter and ii) luciferase, under the control of the HTLV-1 LTR, is activated by Tax [25]. In principle, luciferase, which catalyzes a light emitting reaction in the presence of its substrate D-luciferin, serves as an indirect biomarker for activated NK and T cells in TAX-LUC mice. Alternatively, upon activation of leukocytes during inflammation, neutrophil myeloperoxidases are expressed that catalyze the production of hypochlorous acid from hydrogen peroxide and chloride ions [27]. Luminol emits light when exposed to oxidizing agents and can be used to sensitively and non-invasively detect leukocyte activity during inflammation *in vivo*. We have shown that administration of either luminol or D-luciferin produces bioluminescence in primary TAX-LUC tumors and that microscopic bioluminescent lesions precede tumorigenesis. We sought to determine the effects of inflammation on bioluminescence and tumorigenesis in this model.

**Figure 1:**
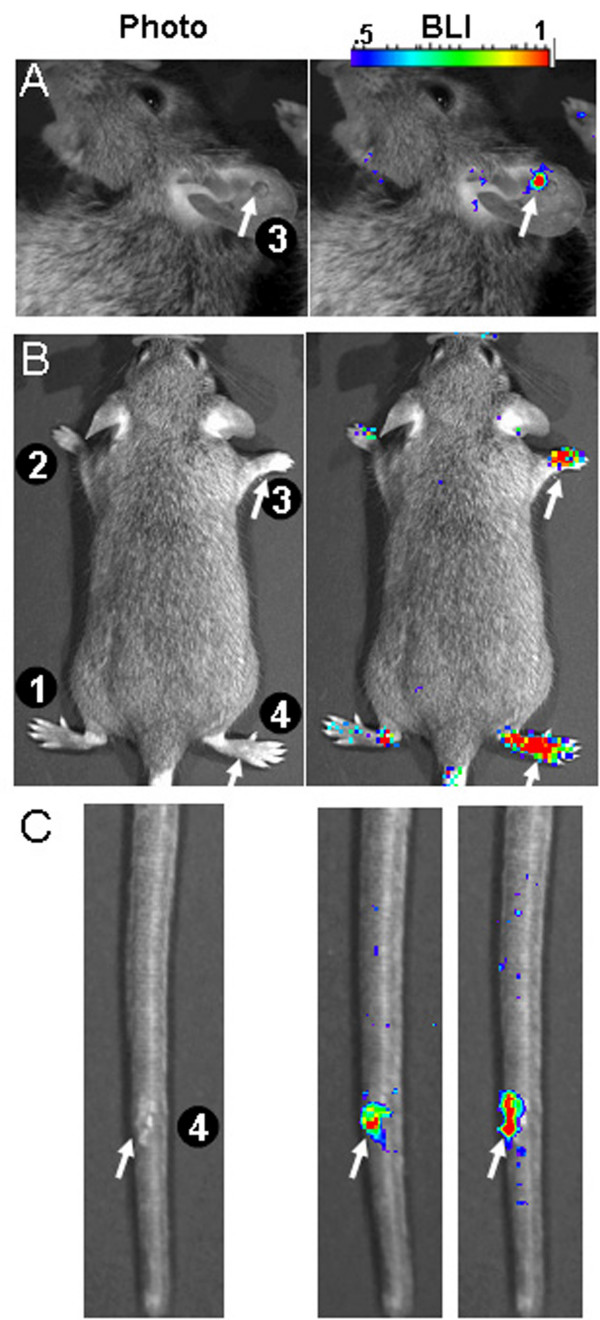
**Wound induced bioluminescence in TAX-LUC mice**. Surgical lesions were experimentally introduced in ear (A) limb (B), and tail tissue (C). The effect of adjuvant on wound associated bioluminescence was also examined (B, C). Treatments include 1) vehicle, 2) CFA, 3) wound, and 4) wound and CFA. Images were obtained 0.5 hrs before treatment, and 0.5, 2, 24, and 48 hrs after treatment. Representative images shown from A) 30 minutes, B) 2 hours, and C) 24 and 48 hours after treatment.

We first asked whether wounding was sufficient to result in a luciferase-mediated bioluminescent signature in TAX-LUC mice. We found that minor incisions on the ear, tail or foot (Fig. 1) were sufficient to produce a significant bioluminescent signature and that introduction of adjuvant in the wound increased the intensity and duration of the signal. These data confirmed a close correlation between wounding and reporter expression *in vivo*.

### Generalized T Cell Activation is Associated with Tumorigenesis

**Figure 2:**
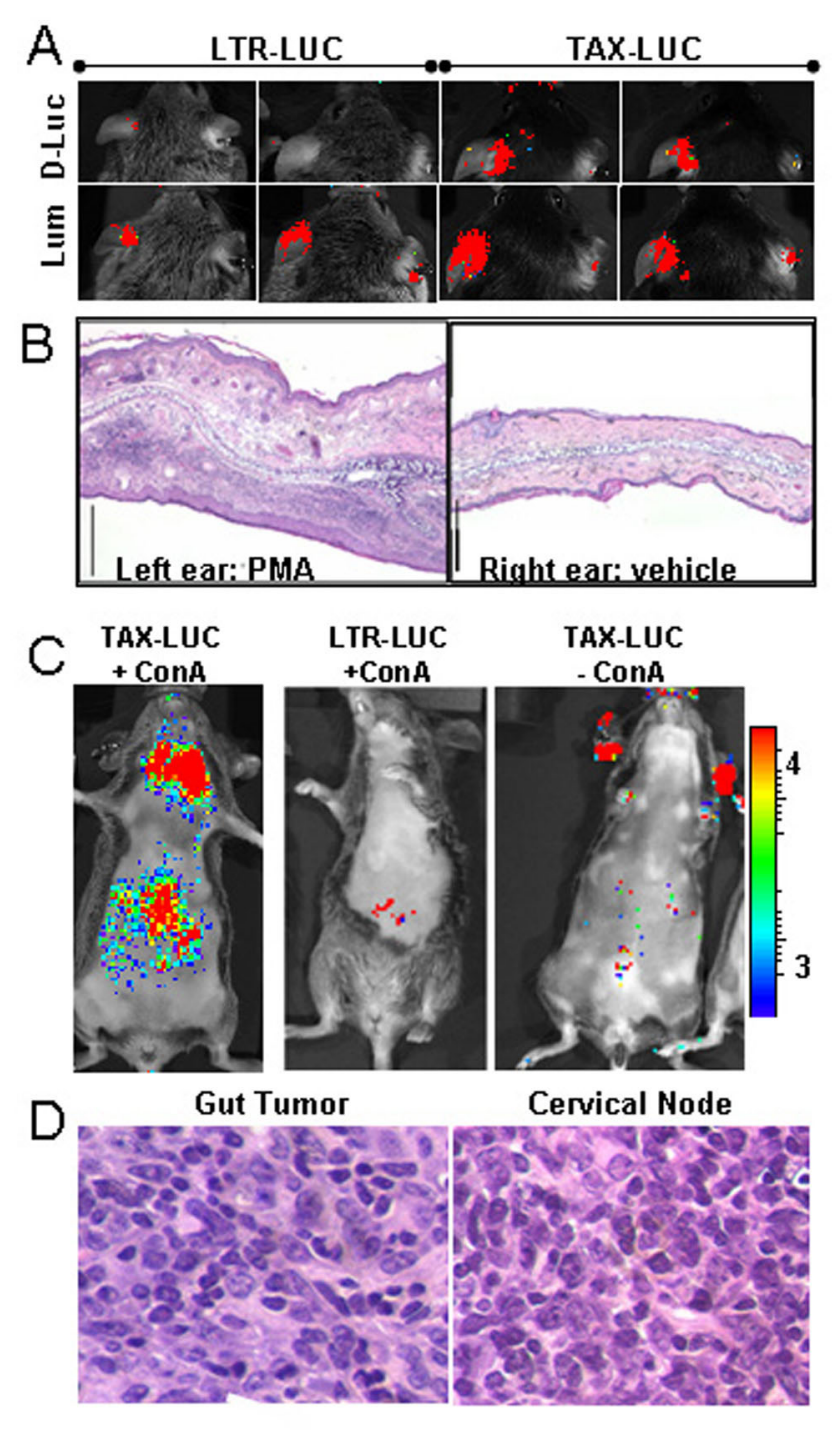
**Phorbol myristyl acetate stimulation of bioluminescence in transgenic mice**. For each mouse, the left ear was treated with PMA and the right ear with vehicle. A) Representative images obtained 2 hours after treatment are shown for two LTR-LUC mice (left panels) and two TAX-LUC mice (right panels) comparing bioluminescence following administration of D-luciferin (top panels) and Luminol (bottom panel). B) Histology showing edema and inflammatory infiltrate associated with topical application of PMA (48 hours; Bar = 1 mm). C) Aggressive lymphoma in TAX-LUC mice from intravenous administration of con A. D) Histology is H/E stained sections of bioluminescent tumors in the cervical lymph nodes and small intestine of a con A treated TAX-LUC mouse.

While Tax is activated in malignant LGL cells of inflamed tumors, the granzyme B promoter is also inducible in T and NK cells by T-cell receptor (TCR)-dependent, TCR-independent, and cytokine-mediated stimuli [28]. A number of direct and indirect inducers of generalized T cell activation were utilized to locally activate this promoter and image Tax activity during inflammation. These included phorbol 12-myristyl 13-acetate (PMA), which when administered topically, promotes T lymphocyte infiltration and activation mediated by protein kinase C, and has been shown to stimulate the human granzyme B promoter in transgenic mice [29,30]. Topical administration of PMA to the ear resulted in luciferase based bioluminescence in TAX-LUC mice, but not LTR-LUC mice (Fig. 2A, top panels) even though a massive inflammatory infiltrate was seen in all PMA treated ears (Fig. 2B). Luminol based bioluminescence emanating from the PMA treated ears compared to the vehicle treated contralateral ears (Fig. 2A, bottom panels) served as a reporter for inflammation. The intensity of luminol BLI after PMA treatment was greater in TAX-LUC mice than LTR-LUC littermates that lack the Tax transgene (fold flux increase 11.5 vs. 7.4; p = 0.018). These findings serve as proof of principle for the appropriate regulation of the transgenes in TAX-LUC mice, confirm that acute inflammation is sufficient to produce bioluminescence in this model, and suggest that Tax expression exacerbates the inflammatory response *in vivo*.

Con A, a potent lectin with broad activity towards T lymphocytes, is also known to activate the granzyme B promoter. To determine whether induction of inflammation affected tumorigenesis in this model, we examined 5 TAX-LUC mice and 5 LTR-LUC in each group given tail vein injections of con A or saline (Fig. 2C). While TAX-LUC mice develop peripheral tumors most frequently on the tail, this method of con A inoculation is known to preferentially target T cell activation in the liver [31,32]. All 5 con A treated mice developed liver bioluminescence, and two died within 1 week of acute hepatitis. The other 3 con A treated mice developed lymphoma initiated in the liver with spread to the gastrointestinal tract, spleen, and cervical nodes, as detected by BLI and histological analysis at necropsy (Fig. 2C). While the 5 saline injected TAX-LUC mice developed tail tumors, none developed a similar form of aggressive lymphoma, characterized by massive visceral infiltration. LTR-LUC animals did not develop tumors. This experiment suggested that con A-induced inflammation and T cell activation in TAX-LUC mice were sufficient to modify the presentation of lymphoma from peripheral and indolent to visceral and aggressive.

**Figure 3:**
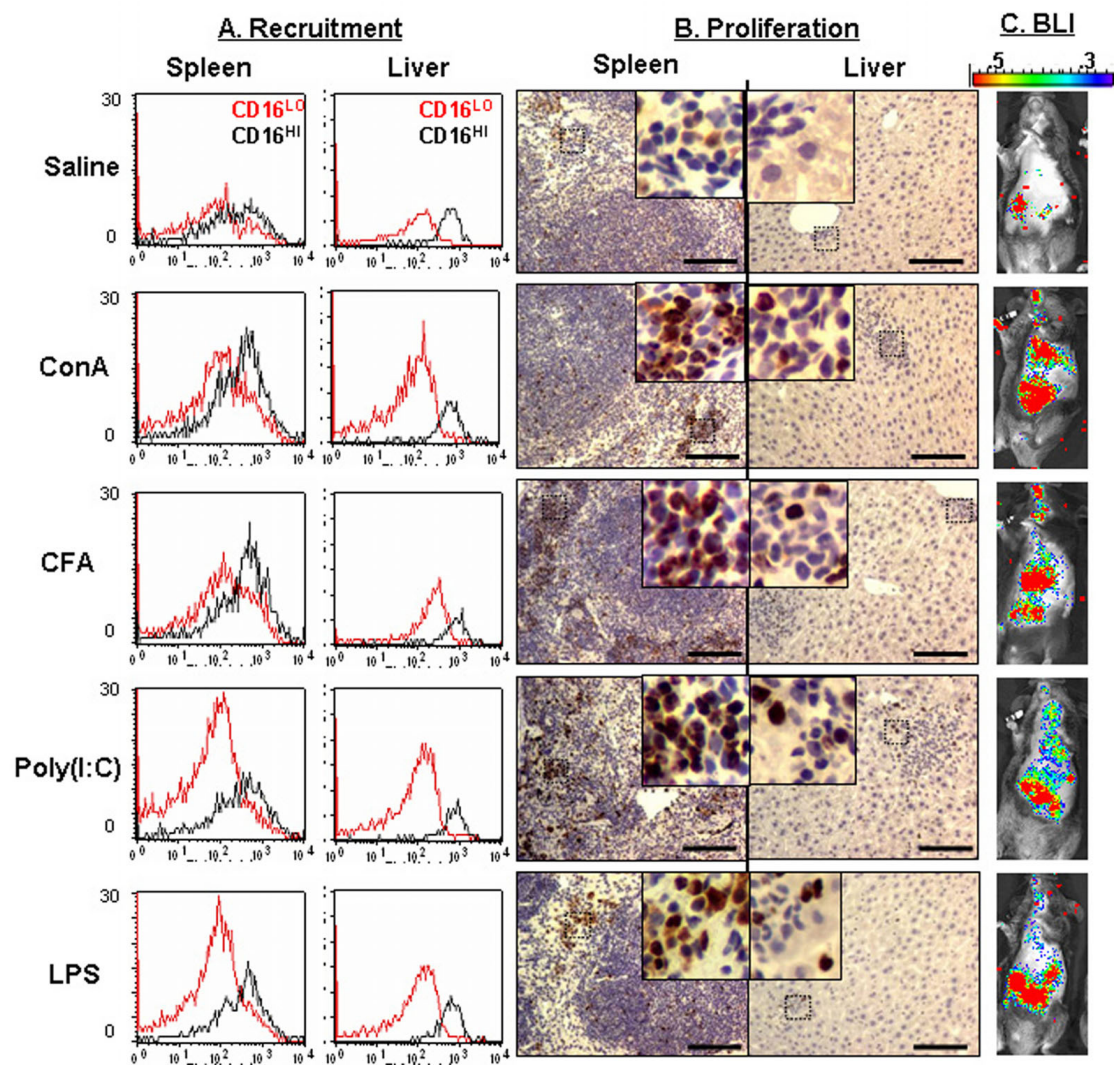
**Bioluminescence in TAX-LUC mice correlates with inflammatory response**. Representative data are shown from groups of 3 mice each inoculated intraperitoneally with saline, con A, CFA, poly (I:C), or LPS. A) FACS histograms for CD16^lo ^cells (red curve) and CD16^hi ^cells (black curve) in liver and spleen 6 hrs after treatment. B) Representative BrdU IHC results from the spleen and liver. C) Bioluminescent images obtained 2 hrs after treatment. Bar = 1 mm.

We also utilized CFA a mixture of paraffin oil, surfactant, and heat-killed mycobacteria that leads to T_H_1 lymphocyte activation [33]. In addition, we examined inducers of T cell activation through effects on TLRs on antigen-presenting cells (APCs). These inducers included poly I:C, a mimic of double stranded RNA that activates the interferon response, and LPS, found in the cell wall of gram negative bacteria, that rapidly activates pyrogenic cytokines and cells involved in innate immunity [34]. In the tumors that arise in TAX-LUC animals, the malignant cells are rarely T cells, but instead are CD16^Hi ^LGLs that lack TCRs. Primary TAX-LUC tumors also contain a large population of CD16^Lo ^cells which are predominantly neutrophils and CD16^- ^cells which include tumor infiltrating T cells. We next sought to determine if bioluminescence resulting from acute inflammation correlates with the recruitment or proliferation of CD16^Hi ^LGLs. The representative results of intraperitoneal injections into 3 mice each of saline, con A, CFA, poly I:C, and LPS are shown in Fig. 3. Mice were imaged 0.5 hour prior to injections and then at 2 and 6 hours after injection, then sacrificed and examined. BLI performed prior to injection exhibited very low background levels of activity primarily within the gastrointestinal tract TAX-LUC mice. Con A treatment resulted in increased numbers of CD16^lo ^cells and BrdU^+ ^cells in the spleen and liver compared to saline treated animals (Fig. 3A, B), whereas the number of CD16^Hi ^cells increased in spleen but not liver. After con A injection BLI was increased in the gastrointestinal tract and liver as compared to saline injected animals (Fig. 3C). Intraperitoneal injection of CFA was similar to the effects of con A. The number of BrdU positive cells in the spleen and liver was increased after CFA treatment, and infiltrates of lymphoid cells in the liver were apparent. Two hours after CFA injection, bioluminescence localized primarily to the liver (Fig. 3C). Intraperitoneal injection of poly(I:C) and LPS also resulted in increased numbers of CD16^lo ^cells and BrdU^+ ^cells in spleen and liver compared to animals injected with saline. Unlike Con A and CFA, bioluminescence in TAX-LUC mice after treatment with poly(I:C) and LPS was more evident in the spleen and gastrointestinal tract than liver.

Taken together, these studies indicated that bioluminescence in TAX-LUC mice serves as a sensitive indicator of acute inflammation *in vivo*. However, the bioluminescence profile does not correlate with CD16^Hi ^cells nor proliferating cells, suggesting the light emitting cells during inflammation are not identical to the population of cells that subsequently undergo malignant transformation. While malignant LGLs in TAX-LUC tumors are bioluminescent, these results demonstrated that during acute inflammation other luciferase-expressing cell types predominate, possibly activated T cells. Based on these findings, we sought to use a genetic approach to determine if activated T cells promote tumorigenesis in TAX-LUC mice.

### Specific T-Cell Receptor Activation Accelerates Tax-Mediated Tumorigenesis

**Figure 4:**
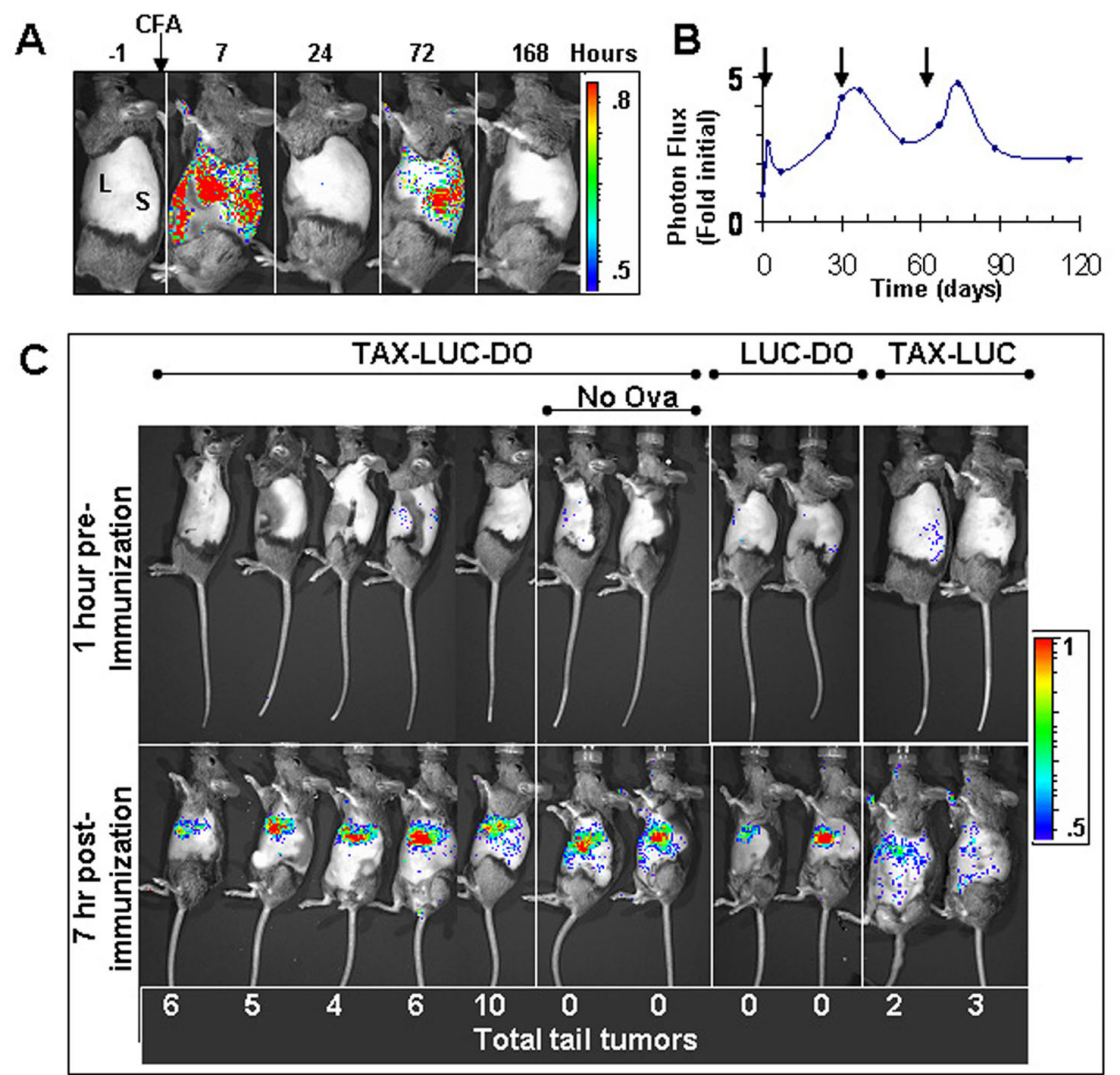
**Bioluminescence imaging of T-cell receptor activation in TAX-LUC-DO mice**. Image (A) and quantitation (B) of the bioluminescence time-course following injections, indicated by arrows in B. C) BL images taken 1 hour prior to and 7 hours after immunization. All animals were injected with CFA and OA except where indicated. The total tail tumors in each animal during the course of the experiment is enumerated at the bottom of the figure.

DO11.10 mice carry a transgenic MHC class II restricted rearranged T cell receptor which reacts with a specific ovalbumin (OA) peptide antigen [6,7]. IP administration of OA results in deletion of immature CD4^+ ^CD8^+ ^TCR^lo ^thymocytes and expansion of CD4^+ ^TCR^Hi ^thymocytes. Within 3 days post injection all of the immature non-OVA reactive thymocytes are removed and OA reactive CD4^+ ^T cells represent approximately 70% of T cells in these mice. In order to examine the specific effects of TCR activation, triple transgenic mice were utilized, resulting from breeding TAX-LUC mice with DO mice (Fig. 4). In one experiment, 5 TAX-LUC-DO mice were inoculated with OA in CFA, and 2 control TAX-LUC-DO mice were inoculated with CFA alone. Double transgenic LUC-DO and TAX-LUC mice were also inoculated with OA in CFA to serve as controls. The immune response to OA in CFA could be observed non-invasively in these mice using BLI (Fig. 4A-B) which served as an internal control to ensure each immunization produced a response. Bioluminescence was detectable 7 hours after injection and by day 3 predominantly localized to the spleen (Fig. 4A). Subsequent injections in primed animals produced a bioluminescent response of increased intensity and duration (Fig. 4B). Interestingly, bioluminescence was also detected in LUC-DO animals, although it was more intense in Tax transgenic animals (Fig. 4C). These results demonstrate that OA in CFA is sufficient to activate basal HTLV LTR transcriptional activity, which is further activated by induction of Tax expression in TAX-LUC-DO mice. Over the course of 1 year, 4-10 tail tumors arose in each of the TAX-LUC-DO mice inoculated with OA in CFA, and 2-3 tail tumors arose in each of the TAX-LUC mice (Fig. 4C, numbers at bottom of panels). No tumors arose in mice lacking the Tax transgene (LUC-DO), nor in the two TAX-LUC-DO controls that received no OA.

**Figure 5:**
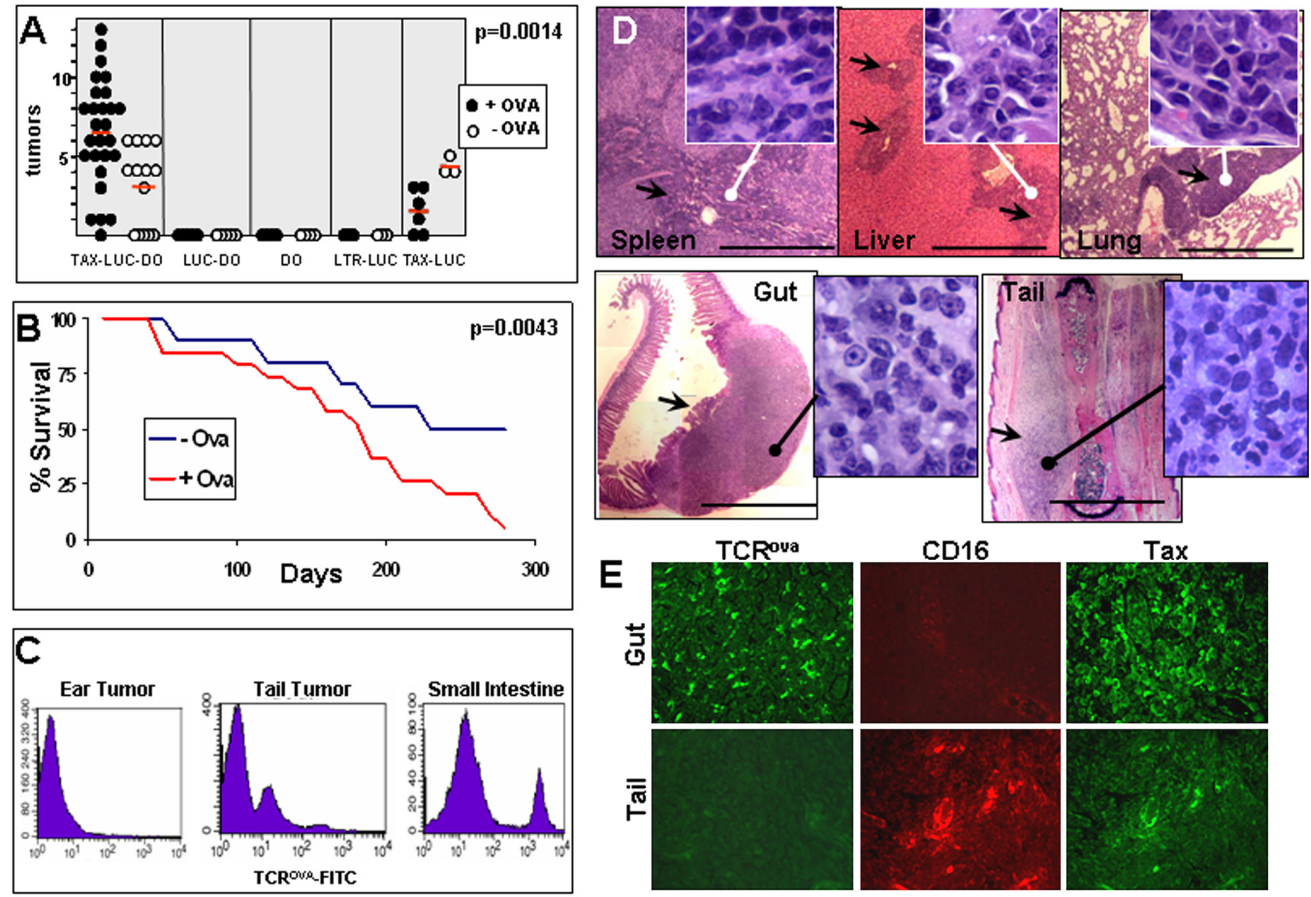
**T-cell receptor activation stimulates tumorigenesis in TAX-LUC-DO mice**. A) Total number of tumors indicated by a single circle for each animal, with closed circles indicating mice immunized with CFA + OA and open circles indicating mice immunized with CFA alone. Red bars indicate the average number of tumors for each group. B) Survival curve for TAX-LUC-DO mice immunized with CFA and OA or CFA alone. C) FACS histograms of TCR expression in tumors that arose on the tail, small intestine, or ear of treated triple transgenics. D) Histology of tumors that infiltrated the spleen, lung, and liver as well as a comparison of gut and tail tumors. E) TCR^ova^, CD16, and TAX expression in tail and gut tumors from a TAX-LUC-DO mouse.

These findings were confirmed and extended in additional experiments (Fig. 5A). Significantly more tumors were noted in triple transgenic TAX-LUC-DO mice inoculated with OA in CFA compared to those inoculated with CFA alone (6.5 vs 3.1, p = 0.0014) (Fig. 5A, panel 1). Moreover, survival was significantly shorter in TAX-LUC-DO and TAX-DO mice treated with OA in CFA compared to those administered CFA alone (Fig. 5B). No tumors developed in the absence of the Tax transgene in LUC-DO mice, DO mice, or LUC mice (Fig. 5A, panels 2, 3, and 4, respectively). Doubly transgenic TAX-LUC mice lacking the specific TCR had fewer tumors in the presence than absence of OA (1.5 vs. 4.3 p = 0.0083). Since the average tumor onset in Tax mice occurs within 200-300 days and many animals do not develop tumors until the second year of life, some Tax positive animals did not develop tumors during the time course of this experiment [18].

While the OA-restricted TCR in TAX-LUC-DO animals is expressed on CD4^+ ^lymphocytes, the presence of TCR^ova ^cells in tumors was variable. Typically, the malignant LGL population in tumors that spontaneously arise in TAX-LUC mice is TCR^-^, and tumor infiltrating lymphocytes are TCR^+^. This is consistent with what we observed in tumors arising on the tails in TAX-LUC-DO mice which included both TCR^- ^and TCR^+ ^cells (Fig. 5C). In contrast, tumors arising in the gastrointestinal (GI) tract, which were only found in TAX-LUC-DO animals treated with OA, were composed of TCR^+ ^cells with a minor population of cells expressing exceptionally high levels of TCR^ova ^(Fig. 5C). Alternatively, tumors arising in the ears contained very few TCR^+ ^cells and were primarily composed of malignant LGLs. Representative histology (Fig. 5D) for tumors arising in OA stimulated TAX-LUC-DO mice, includes examples of tumors invading spleen, lung, and liver as well as primary tumors arising in intestine and peripheral tissues. In each case, a proliferation of lymphoid cells is evident, however, the size, morphology, and expression profiles of CD16 and TCR^ova ^indicated that tumors arising in the gut were distinct from peripheral tumors that typically arise on TAX-LUC mice. Unlike peripheral tumors arising in the tail or ear, gut tumors include very few if any CD16 expressing cells but an abundance of TCR^ova ^+ and Tax expressing cells (Fig. 5E). Taken together, these results indicate that T cell activation in TCR transgenic TAX-LUC mice resulted in increased peripheral tumor burden, decreased survival, and the presentation of a novel form of visceral lymphoma composed of CD16^- ^TCR^ova ^lymphocytes similar to tumors that arose in con A treated TAX-LUC mice.

## Discussion

Cells within an inflammatory microenvironment are capable of promoting malignancy. The cell types involved in this process, and the mechanisms by which it occurs have not been fully characterized. While T cells are recruited to sites of chronic inflammation and are present in many tumors, they have been shown to have varied roles in the regulation of cancer. CD8^+ ^cells may play a role in restricting neoplasms through direct cellular cytotoxicity or release of cytokines or chemokines [35]. CD4^+^CD25^+ ^T_reg _cells repress inflammation, but have been found to be elevated in several different human cancers, and suppress immune responses [5]. CD4^+ ^T_H_17 cells, that secrete IL-17, have been shown to accumulate in the tumor microenvironment and contribute to the pathogenesis of cancers [8]. Which of these competing activities dominates the microenvironment of a chronically inflamed tumor *in vivo*? We sought to determine if activated T- cells repress or promote tumor growth in a mouse model of inflammation associated cancer. For these studies, we have used several different forms of general or specific T- lymphocyte activation and in our experimental model we found that activated T- cells in the context of inflammation strongly favor a tumor promoting environment. In the animal model we used, Tax transgenic tumors are characterized by constitutive NF-kB activity, expression of IL-1, IL6, TNF-α, and GM-CSF, severe neutrophilia, and marked osteolytic activity, all of which are also associated with T_H_17 activity [20,21,24,26]. IL-1 and IL-6 produced by tumor cells, fibroblasts, and APCs are potent in expanding memory T_H_17 cells [9]. IL-17 promotes expansion and recruitment of neutrophils and cooperates with TLR ligands to enhance inflammatory reactions [10]. While IL-17 is not expressed by the malignant LGL cells that arise in TAX-LUC tumors, it is elevated in the serum of tumor-bearing mice. The role of T_H_17 cells in promotion of early events in inflammation-associated tumorigenesis in this model will be the focus of future studies.

The following model is consistent with information available to date. Tumorigenesis in TAX-LUC mice begins as a microscopic intraepithelial lesions associated with activated neutrophils, detected with luminol, and oncogene expression, measured by luciferase activity. Among the inflammatory cells attracted to sites of wounds, neutrophils arise first, followed by mast cells and monocytes, which differentiate into macrophages. It is of interest that wounding has previously been found to be critical for tumorigenesis in *v-jun *transgenic mice [36]. The next step in tumorigenesis in this model results from the ability of Tax to directly and indirectly mediate constitutive activation of both the canonical and non-canonical pathways of NFkB. This prevents apoptosis and promotes proliferation of Tax expressing LGL cells that have been recruited to the wound [13]. The third step in our model is genetic instability also catalyzed by Tax. Both NFkB activity and genetic instability are associated with cancers unrelated to HTLV-1 disease. In our model Tax is simply a mechanism to accomplish these activities in an accelerated manner *in vivo*. The fourth step in TAX-LUC tumor development, the focus of this work, is the activation of T- cells that have also been recruited to the wound. Activated T-cells release cytokines and chemokines, promote induction of angiogenesis, and regulate the immune response via direct cell contact and activation of macrophages, dendritic cells, and neutrophils. The resulting cytokine storm exerts systemic effects with a broad range of biological consequences. Neutrophil infiltration into Tax transgenic tumors is prominent, and is often accompanied by peripheral blood neutrophilia [18,26]. Neutrophils may promote tumor cell proliferation directly. Alternatively, myeloid-derived suppressor cells have been described which inhibit anti-tumor immunity [37]. It is noteworthy that adjuvant-induced inflammation alone was not sufficient to promote tumorigenesis in TAX-LUC or TAX-LUC-DO mice. The addition of OVA to stimulate the T-cells was required for the phenotype, indicating a critical role for T-cells in this step. This model of tumorigenesis for inflammation associated cancers is consistent with the data currently available and leaves open many avenues of further inquiry.

Although alternative Tax transgenic models have been described, only two other models were characterized by enhanced T- cell proliferation [38-40]. The role of inflammation in those model systems remains to be assessed. We are currently developing new transgenic lines to pursue these lines of inquiry including TAX-LUC mice in which i) Tax activity can be experimentally regulated in an inducible expression system, ii) NFkB signaling is restricted, or iii) cytokines critical for development or activation of T- or NK- cells are absent. We propose that the answers to these questions will have broad implications to cancers associated with similar mechanisms of origin.

## Conclusions

Bioluminescent imaging with HTLV-1 Tax transgenic mice provided a sensitive marker of inflammation and tumor formation. Use of this model demonstrated that wounding, T- cell activation, and exposure to chemical agents exacerbated development of lymphoma.

## Competing interests

The authors declare that they have no competing interests.

## Authors' contributions

DR, ML, DPW, and LR have made substantial contributions to conception and design. DR, SG, JH, and SN have made contributions to data acquisition. DR, SG, JH, DPW, and LR have made contributions to data analysis. DR and LR have been involved with drafting the manuscript.
